# Preparation, characterization and reusability efficacy of amine-functionalized graphene oxide-polyphenol oxidase complex for removal of phenol from aqueous phase

**DOI:** 10.1039/c8ra06364h

**Published:** 2018-11-14

**Authors:** Pravin M. D., Chris Felshia S., A. Gnanamani

**Affiliations:** Biological Material Laboratory (Microbiology Division), CSIR-CLRI Adyar Chennai 20 Tamil nadu India gnanamani3@gmail.com

## Abstract

The present study explores the preparation, characterization and reusability efficacy of an amine-functionalized graphene oxide and polyphenol oxidase complex for the removal of phenol from aqueous phase. In brief, graphene oxide (GO) is synthesized according to modified Hummer's method using graphite powder and functionalized with amine using the Bucherer's method (GO-NH_2_). Partially purified polyphenol oxidase (PP-PPO) enzyme extracted from *Solanum tuberosum* is used for the preparation of the complex. The resultant GO-NH_2_-(PP-PPO) complex is used for the phenol degradation studies. The samples of GO, GO-NH_2_, and GO-NH_2_-(PP-PPO) complex are characterized using various instrumental techniques. Spectral UV data and FTIR and XRD diffraction patterns confirm the amine functionalization on GO. Raman spectrum, SEM micrograph and thermogravimetric (TG) analyses authenticate the linked enzyme on GO-NH_2_. GO-NH_2_-(PP-PPO) complex demonstrates >90% enzyme stability at all the studied temperatures (4 °C, −20 °C, RT and 37 °C). Phenol degradation studies show >99% removal of 1000 ppm of phenol within 5 hours from the start of the experiment at the optimized pH of 5.0 and temperature of 30 °C, as inferred from HPLC analysis. Catechol and hydroquinone compounds are identified as intermediates during the removal of phenol. Furthermore, studies on the reuse of GO-NH_2_-(PP-PPO) complex suggest that the complex can be used effectively for the removal of phenol up to maximum 7 cycles. In conclusion, the observations made in the present study show that the complex containing amine-functionalized graphene oxide and phenoloxidase is effective for the removal of phenol with appreciable reusability.

## Abstract 2

Schematic representation of the present study describing amine functionalization followed by enzyme immobilization and degradation of phenol.

## Graphical abstract

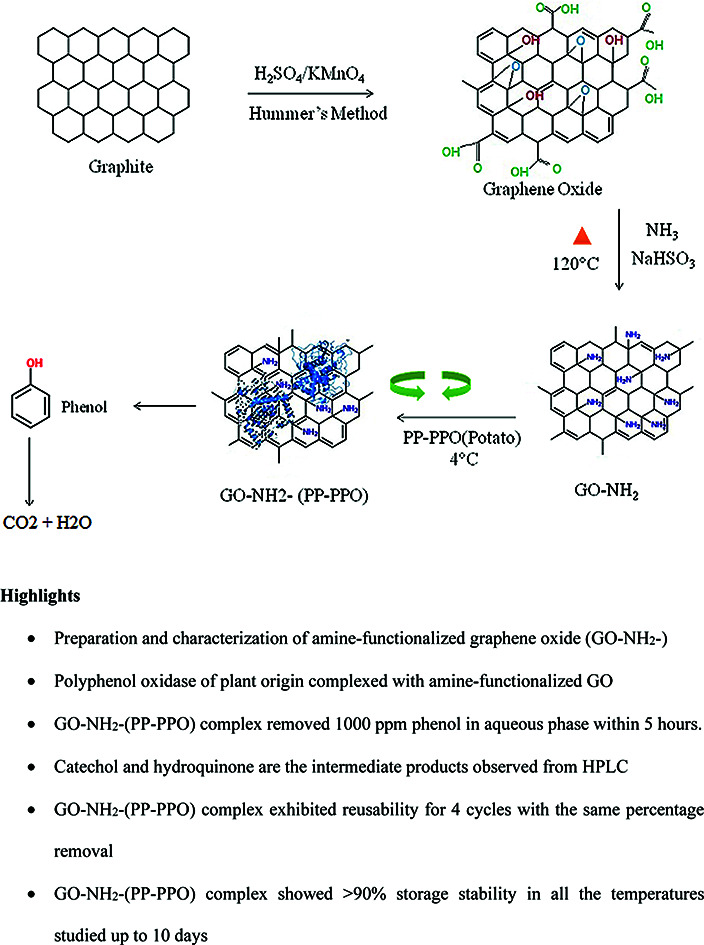

## Introduction

Increasing environmental pollution in the past few decades has caused adverse effects on the quality of life. Phenol and its derivatives are among the most common air and water pollutants and are of concern due to their toxicity.^1^ Pharmaceutical and medical industrial processes release a large quantity of wastewater containing phenol at the concentration as high as 10 g L^−1^.^2^ Phenol is a priority pollutant, as termed by US EPA, and the acceptable limit for phenolic compounds in drinking water is 1 μg L^−1^.^3^ Wastewater containing phenol and its derivatives needs adequate treatment before its release into the environment, thus creating a necessity for the development of various physical, chemical and biological treatment methods.

Microbial degradation of phenol has been the focus of research of the scientific community over the past few decades with an emphasis on the degradation of high concentrations of phenol.^4^ The ability of microorganisms to mineralize pollutants mainly depends on specific enzymes. Enzymatic degradation and detoxification of phenol are advantageous because of its high substrate specificity, high catalytic efficiency, and mild reaction conditions.^5^ Among the enzymes used for wastewater treatment, polyphenol oxidases (PPO), belonging to the class oxidoreductases, catalyze the transformation of phenolic and non-phenolic aromatic compounds.^6^ Laccases and tyrosinases are subgroups of polyphenol oxidase, utilizes molecular oxygen to convert phenolic compounds to quinones.^7,8^ Both enzymes are metallic proteins with copper in their catalytic sites.^9,10^ Mukherjee *et al.*^11^ comprehensively described the remediation of phenol using PPO, an enzyme found naturally in apples, bananas, grapes, mushrooms, lettuce and other fruits.

Although the use of enzymes in biotechnology and environmental applications has increased tremendously, enzymes have certain shortcomings such as lack of long-term operational stability, difficulties during recovery, reusability, sensitivity to denaturing agents and adverse sensory and toxicological effects, which hinder their use in environmental applications.^12^ Immobilization of an enzyme on a solid support can circumvent these shortcomings. Immobilization increases stability, catalytic activity, recovery, and reusability and reduces susceptibility to microbial contamination when compared to that for free enzymes.^13,14^ Immobilization of PPO increases the efficiency in the treatment of wastewater compared to that for free enzymes.^15,16^ Enzyme immobilization matrices are not restricted to calcium and copper alginate and polyamide membranes, cinnamoylated derivative-coated glass beads, chitosan beads, Celite, cellulose and Eupergit carriers, and magnetic mesoporous silica nanoparticles.^17–21^

Graphene oxide (GO) may serve as an immobilization platform for enzymes *via* noncovalent adsorption and covalent binding. Graphene oxide (GO) exhibits a two-dimensional, sp^2^-hybridized, hexagonal lattice with oxygen-containing functional groups.^22,23^ The active surface of GO provides many reaction sites for linking the external species such as small molecules, polymers, proteins, biomacromolecules, and inorganic nanoparticles.^24,25^ Zhang *et al.*^26^ described GO as an ideal solid substrate for enzyme immobilization. However, the use of GO for immobilization depends on the surface chemistry of GO, intrinsic properties of the enzyme and the immobilization method.

Immobilization methods include adsorption, cross-linking encapsulation, entrapment and covalent binding. Apart from these methods, direct enzyme immobilization on a GO surface without any coupling reagents has been reported.^27,28^ However, enzymes directly linked to the surface of GO are susceptible to environmental changes, such as pH and temperature, and their functional groups can make immobilization unfavorable if electrostatic repulsion occurs.^29^ For example, the activity of chymotrypsin can be strongly inhibited by the use of as-made GO as an immobilization matrix, which necessitates the functionalization of GO to make it suitable for enzyme immobilization.^30^

Amine-functionalized GO might serve as an appropriate material to complex with enzymes.^31^ The nucleophilic nature of amine's nitrogen atom is speculated to increase the interfacial binding between GO and the material of interest.^32^ Amine functionalization enhances covalent bonding to polymers and biomolecules in carbon nanotubes.^33^ Though functionalization affects enzymatic activity in most cases, thermal stability, storage stability, and reusability of the enzyme immobilized on amine-functionalized GO sheets as a complex may improve significantly.^34^ Thus, the aim of the present study is to prepare a complex with partially purified polyphenol oxidase (PPO) extracted from *Solanum tuberosum* on amine-functionalized graphene oxide (GO) using covalent bonding and to evaluate phenol degradation efficiency as well as storage stability and reusability of the GO-NH_2_-(PP-PPO) complex in detail.

## Experimental methods

### Synthesis of graphene oxide

Graphene oxide (GO) was synthesized using modified the Hummer's method in an ice bath.^35^ In brief, 0.5 g of graphite flakes and 0.25 g of NaNO_3_ were homogenized in the presence of 11 mL conc. H_2_SO_4_ (98%). The mixture was then stirred for 1 h, after which 3 g of KMnO_4_ was added and stirring was continued. After 1 h, 23 mL of water was added, and the mixture was stirred for an hour. The mixture was then diluted by the addition of 40 mL of water and finally mixed with 2.5 mL of H_2_O_2_. The resulting solution was centrifuged at 7500 rpm and 1 N HCl was added in the ratio of 1 : 10. The resulting precipitate was washed repeatedly with distilled water until the pH reached 7.0; then, it was dispersed in water and used for experimental studies.

### Functionalization of graphene oxide

The synthesized GO was functionalized with amine (–NH_2_) groups using modified Bucherer's method.^32^ In brief, 0.1 g of GO was dispersed in 100 mL of water (1 mg mL^−1^) under sonication for 30 min to get a uniform suspension. To 10 mL of the dispersed GO solution, 10 mL of 1 N ammonia and 10 mL of 1 N sodium bisulfite were added. The resulting mixture was incubated at 120 °C for 1 h and centrifuged. The pellet was dried at 60 °C and used for experimental purposes.

### Polyphenol oxidase

Polyphenol oxidase (PPO) was extracted from *Solanum tuberosum* according to the procedure described elsewhere. In brief, fresh potatoes (100 g) were washed and cut into pieces and homogenized with the equal volume of sodium fluoride. Ammonium sulfate (60 g) was added to the homogenized solution and the mixture was incubated overnight at 4 °C. The resulting solution was centrifuged at 10 000 rpm for 15 min and the pellet obtained was dissolved in 10 mL of citrate buffer (pH 4.8). The buffer solution was centrifuged at 10 000 rpm for 15 min; the collected supernatant was subjected to dialysis for 48 h using standard dialysis procedure, and the product was lyophilized. The lyophilized powder obtained was designated as partially purified PPO (PP-PPO) and stored at 4 °C for further use. The activity of PP-PPO was measured during each step of extraction to ensure the stability of the enzyme.

### Activity of PPO (polyphenol oxidase)

The procedure for the determination of polyphenol oxidase activity is as follows. A mixture of 2.0 mL 50 mM phosphate buffer (pH 6.5), 0.6 mL of distilled water and 0.2 mL of 50 mM catechol was prepared. To this mixture, 0.2 mL of the test enzyme solution was added and the absorbance was measured at 410 nm using a UV-visible spectrophotometer. Distilled water was used in the place of catechol for a blank solution. All the experiments were performed in duplicates. The activity of the enzyme was calculated using the following formula:1
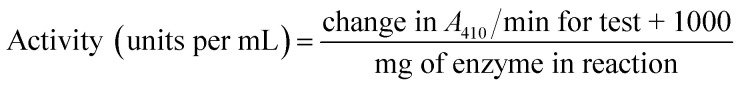


One unit of enzyme activity is defined as the amount of enzyme that catalyzes the conversion of 1 mM of catechol per minute.

### PP-PPO and GO-NH_2_ complex preparation

PP-PPO has been complexed with amine-functionalized GO to form GO-NH_2_-(PP-PPO) complex. In brief, 10 mg of lyophilized enzyme was mixed with 30 mL of GO-NH_2_ solution (1 mg mL^−1^ concentration) and stirred for 1 h at 4 °C. The solution was then centrifuged at 10 000 rpm for 15 min. The amount of unbound enzyme was analyzed by evaluating the protein concentration of the supernatant using Lowry's method.^36^ The loading capacity of GO-NH_2_ was determined according to the following formula:2
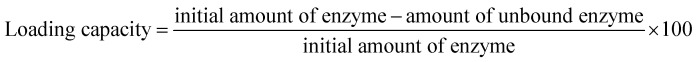


The samples were lyophilized and characterized using analytical techniques.

### Characterization studies

The synthesized GO, GO-NH_2_, and GO-NH_2_-(PP-PPO) complex samples were characterized using various analytical and imaging techniques. The samples were suspended in aqueous medium and analyzed using a UV spectrophotometer in the range from 200 to 400 nm (Jasco, UV spectrophotometer 2450, Japan). The structural analysis of the samples was performed using XRD at diffraction angles (2*θ*) from 5 to 80° for GO, GO-NH_2_ and GO-NH_2_-PPO. KBr pellet technique was used to prepare samples for FTIR, and FTIR spectra were recorded for GO, GO-NH_2_ and GO-NH_2_-(PP-PPO) samples in the range of 400–4000 cm^−1^. Confocal Raman system equipped with Nd:YAG laser was used for excitation of GO, GO-NH_2_ and GO-NH_2_-(PP-PPO). The morphology of GO, GO-NH_2_ and GO-NH_2_-(PP-PPO) samples was imaged using a Phenom Pro Scanning Electron Microscope. The zeta potential of GO, GO-NH_2_ and GO-NH_2_-(PP-PPO) was analyzed using a zeta analyzer. The thermal stability of GO, GO-NH_2_ and GO-NH_2_-(PP-PPO) was analyzed using a Q-50 Thermo analyzer (TGA) in the range of 20–800 °C under a constant nitrogen flow (60 mL min^−1^).

### Phenol degradation studies using free PP-PPO and GO-NH_2_-(PP-PPO) complex

Degradation experiments were carried out using both free enzymes and the GO-NH_2_-(PP-PPO) complex at pH 7.0 and 30 °C, and the degradation efficiency was calculated with respect to the incubation period. Optimization studies were carried out for pH in the range of 4.0–9.0 and temperature in the range of 30–80 °C. Furthermore, the stability of the immobilized enzyme at various pH and temperatures was determined accordingly. In a typical experiment, 1000 ppm of phenol was incubated with 30 mg of GO-NH_2_-(PP-PPO) complex for the scheduled time period of 1–5 hours. Samples were withdrawn at scheduled time intervals and subjected to HPLC analysis after centrifugation and 0.45 μm membrane filtration. The analytical parameters for HPLC analysis were as follows: C-18 column, absorbance measurement at 280 nm, flow rate of 1.0 mL min^−1^, methanol and water mixture as mobile phase (v/v 50 : 50). The reduction in phenol concentration was calculated based on the standard graph constructed using phenol of different concentrations.

### Reusability

The GO-NH_2_-(PP-PPO) complex exposed to phenol was reused, and the degradation potential of the reused enzyme complex was assessed. In brief, GO-NH_2_-(PP-PPO) sample incubated with phenol was centrifuged after a scheduled time interval and the supernatant was removed. The pellet was then washed three times with phosphate buffer (pH 6.5) and incubated with fresh phenol (1000 ppm) at an optimum temperature for 300 min. This procedure was repeated ‘*n*’ number of times until the degradation potential decreased significantly. The relative efficiency was calculated using the following formula:3
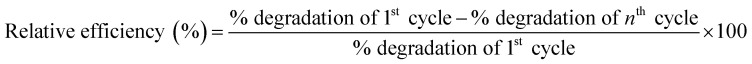


### Storage stability

The storage stability of the GO-NH_2_-(PP-PPO) complex was assessed by incubating the samples at four different temperatures (−20, 4, 25 and 37 °C) for the period of 30 days, and the phenol degradation potential was assessed at scheduled time intervals of 10 days.

## Results and discussion

As described in the introduction, pollutant removal from an aqueous phase necessitates an advanced and improved method. The current research scenario for graphene suggests its potential for pollutant removal. However, graphene's potency has further been improved by altering its surface charge and energy through functionalization, which facilitates the immobilization of degradative enzymes. Compared to free enzymes, immobilized enzymes play a major role in minimizing the use of enzyme and preventing the loss of enzyme activity. Though numerous research methods are in public domain on enzyme immobilization, there is a demand for a suitable matrix for enzyme immobilization due to the requirements of possible reuse for multiple cycles. In this context, graphene oxide (GO) is considered as an appropriate matrix. However, to have an appreciable percentage of the immobilized enzyme, plain GO needs surface modifications. In recent times, amine-functionalized graphene oxide exhibits applications in water treatment.^37^ Hence, in the present study, GO obtained from graphite was further functionalized with amine, and the resultant GO-NH_2_ was used for enzyme immobilization. Here, the enzyme chosen is polyphenol oxidase (PPO), which is known for its pollutant removal under free enzyme conditions as well as under immobilized conditions. Since phenol has been selected as the pollutant, PPO can be a suitable enzyme for the study. Furthermore, the source of PPO also plays a major role in designing experiments for field scale levels. Potato PPO is a chief and a suitable source based on its availability and the extraction technique. For each experimental step, necessary characterization studies of GO, GO-NH_2_, and GO-NH_2_-(PP-PPO) complex samples were performed to authenticate the expected output and to remove phenol.

**Fig. 1:**
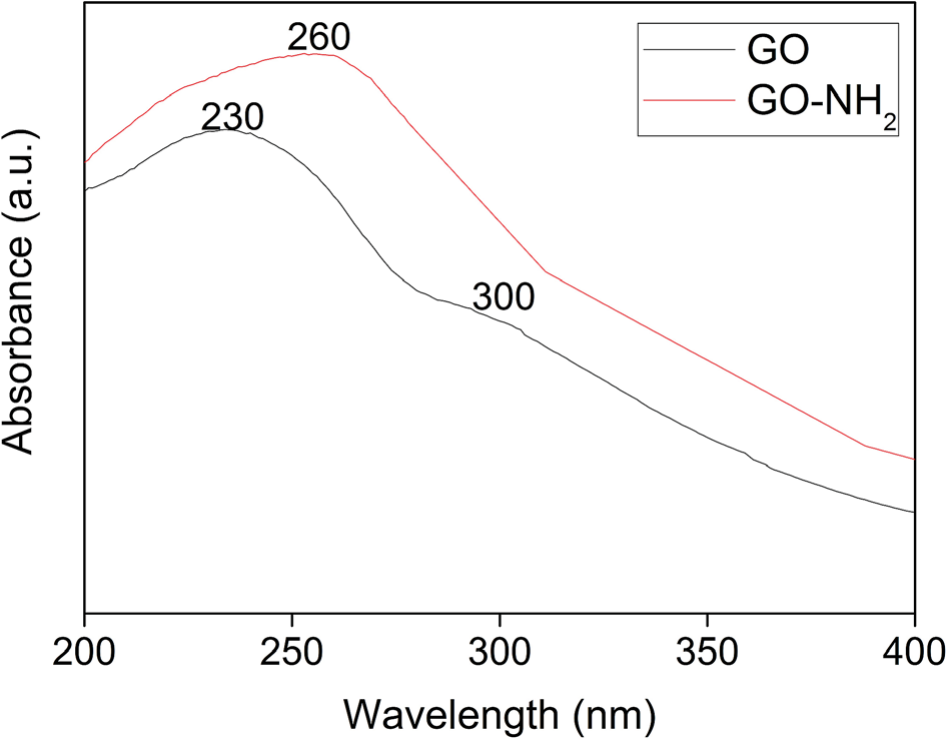
UV-visible spectra of graphene oxide (GO) synthesized from graphite and amine-functionalized graphene oxide (GO-NH_2_).

Fig. 1 illustrates UV-visible spectra of GO and GO-NH_2_. The peak at 230 nm and a shoulder in the 300 nm region for GO correspond to the π–π* transition of aromatic C–C bonds and the n–π* transition of C=O bonds, which are similar to the results of a previous report.^38^ Upon amine functionalization, a redshift from 230 nm to 260 nm is observed, and the disappearance of a shoulder at 300 nm suggests the replacement of phenolic oxygen atoms by NH_2_ functional groups on the surface and the edges of GO during Bucherer's reaction.^39^ After complexation of PP-PPO, an insignificant shift in the absorption peak at 260 nm and a shoulder at 280–300 nm confirm the immobilization of enzyme on to the GO-NH_2_ matrix.

**Fig. 2:**
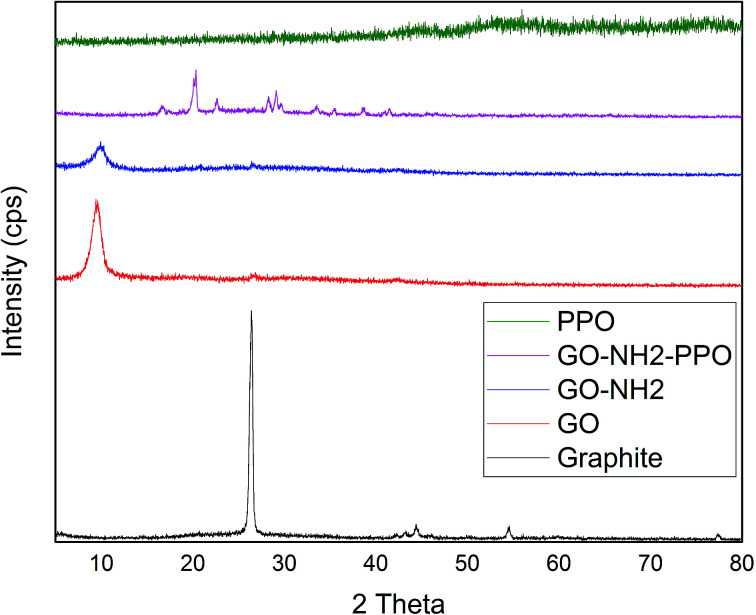
XRD patterns of graphite, GO (graphene oxide), GO-NH_2_ (amine-functionalized graphene oxide) and GO-NH_2_-(PP-PPO) (amine-functionalized graphene oxide partially purified polyphenol oxidase enzyme) complex.

Fig. 2 illustrates the XRD patterns of graphite, GO, GO-NH_2_ and GO-NH_2_-(PP-PPO) complex samples. The XRD pattern of graphite shows a sharp diffraction peak at 26.4° with *d*-spacing of 3.35 A°, which represents the characteristic (002) plane peak of hexagonal graphite (JCPDS number 75-1621). After chemical exfoliation, the synthesized GO shows a sharp peak at 10.05° with *d*-spacing of 8.79 A°. The larger *d*-spacing is ascribed to the addition of oxygen during chemical exfoliation of graphite, resulting in the formation of hydroxyl, epoxy and carbonyl functional groups.^40^

Upon amine functionalization, *d*-spacing further increased to 9.35 A°, which indicated that the surface is functionalized with NH_2_ groups. According to Navaee and Salimi,^32^ the increase in *d*-spacing helps immobilization of enzyme on GO. After immobilization of PP-PPO onto GO-NH_2_, the diffraction peaks of GO disappeared completely, and new peaks were observed at 16.59°, 20.27°, 22.42°, 28.25°, 29.22°, 33.30°, 38.75°, 40.00° and 43.36°. The diffraction peak observed for graphite matched the one reported by An Gong *et al.*^41^ and Caliman *et al.*^42^ Upon transformation to GO, the peak shifted to a lower region, which is consistent with the results reported by the abovementioned authors. Furthermore, after amine functionalization, the intensity of the peak at 2*θ* was reduced, indicating that amine groups were introduced between the layers of graphene oxide. According to Caliman *et al.*,^42^ the introduction of amine groups into the GO structure accompanied by partial reduction of other functional groups with the interlayer spacing remaining practically unchanged corroborates our findings. The XRD pattern of the enzyme alone indicated amorphous nature despite the presence of copper in the active sites. However, when it was immobilized with amine-functionalized graphene, interestingly, peaks with lower intensity observed at higher angles (30–45°) may be due to the change in interlayer spacing. Moreover, the diffraction peaks observed between 30 and 40° partially matched the diffraction pattern of copper and copper oxide, thus supporting the occurrence of immobilization. According to An Gong *et al.*,^41^ immobilization of the enzyme naringinase increases the crystalline sizes of graphene particles, which is reflected in the intense peak at 2*θ* theta value.

**Fig. 3:**
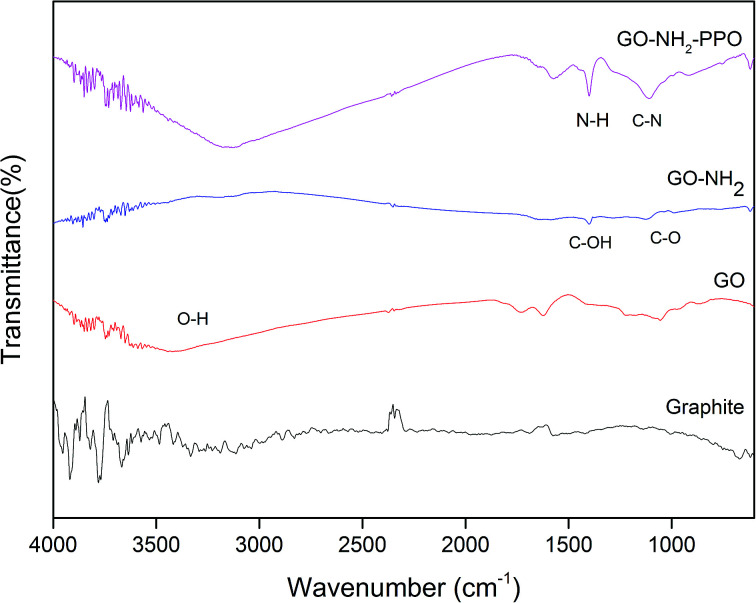
FTIR spectra of graphite, GO, GO-NH_2_ and GO-NH_2_-PPO samples.

**Fig. 4:**
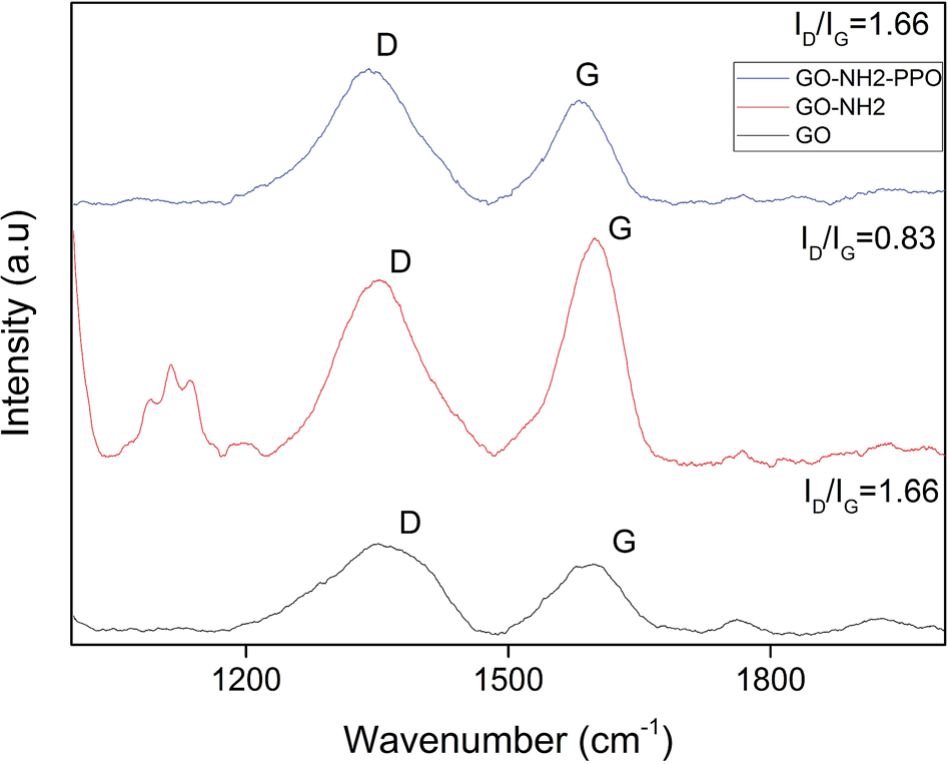
Raman spectra of GO (graphene oxide), GO-NH_2_ (amine-functionalized graphene oxide) and GO-NH_2_-(PP-PPO) (amine-functionalized graphene oxide partially purified polyphenol oxidase enzyme) complex.

FTIR spectra of GO, GO-NH_2_ and GO-NH_2_-(PP-PPO) complex validate the functionalization of NH_2_ on the surface of GO (Fig. 3) as well the enzyme. FTIR spectra of GO showed the presence of C–H rock (860 cm^−1^), C–H “oop” (980 cm^−1^), alkoxy/alkoxide C–O stretches (1050 cm^−1^), epoxide/ether C–O (1217 cm^−1^), C–H rock (1370 cm^−1^), aromatic ring C–C in-ring stretch (1630 cm^−1^) and C=O stretching vibration (1726 cm^−1^). The broad peak corresponding to O–H stretching vibration in GO (3405 cm^−1^) disappeared in the FTIR spectrum of GO-NH_2_. Two new peaks were observed at 3030 and 3200 cm^−1^, corresponding to N–H stretching of primary/secondary amides. The peaks at 1596 and 1650 cm^−1^ corresponded to the N–H bending of primary amines, and the peak at 1030 cm^−1^ corresponded to N–H wag of primary/secondary amines, indicating the presence of amine groups. The intensity of the C–O and C=O peaks decreased, indicating the removal of oxygen groups. Since there is no covalently bonded nitrogen on GO, the C–N stretch peak at 1160 cm^−1^ indicated the functionalization of amine on the surface of GO during Bucherer's reaction.^43^ The FTIR spectrum of GO-NH_2_-(PP-PPO) complex showed the presence of a broad peak at 3130 cm^−1^, corresponding to the N–H vibration of PP-PPO. The peaks at 1658 and 1571 cm^−1^ corresponded to primary and secondary amide groups of PP-PPO.^44^ The C–C peak and C–H peak at 1430 cm^−1^ and 1330 cm^−1^ for GO-NH_2_ and at 1405 and 1300 cm^−1^ for GO-NH_2_-(PP-PPO) corresponded to stretching and rocking peaks of graphene oxide. The peak at 920 cm^−1^ corresponded to N–H wag of primary/secondary amines, and the peak at 1112 cm^−1^ corresponded to C–N stretch due to amine functionalization on the surface of GO. Raman spectra (Fig. 4) further supported amine functionalization and immobilization of enzymes.

The graphitic G band related to E_2g_ vibration mode of sp^2^ carbon domains was observed at ∼1584 cm^−1^ for GO, 1594 cm^−1^ for GO-NH_2_ and 1599 cm^−1^ for GO-NH_2_-(PP-PPO), which explains the degree of graphitization. The intensity of D band explains the structural defects in graphitic structure and partially disordered structures of sp^2^ domains. The D band observed at 1340 cm^−1^ shifted to 1354 cm^−1^ for GO-NH_2_ and GO-NH_2_-(PP-PPO) complex. The defects introduced by oxidation of graphite are clear from the *I*_D_/*I*_G_ ratio of 1.28 for GO, indicating the change in hybridization from sp^3^ to sp^2^.^45^ Upon functionalization of amine groups, the *I*_D_/*I*_G_ ratio increased from 1.28 to 1.31. The *I*_D_/*I*_G_ ratio is a measure of disorderliness and describes the relative disorder due to structural imperfections. Enhanced defects due to sp^3^ hybridized C atoms might be responsible for the increase in *I*_D_/*I*_G_ ratio after functionalization. The *I*_D_/*I*_G_ ratio decreased substantially upon enzyme immobilization. The possible reason for this observation might be the inherent carbon vibration of the enzyme increasing the intensity of G band. The new peaks arising around 1100 cm^−1^ correspond to those of the PP-PPO enzyme.

**Fig. 5:**
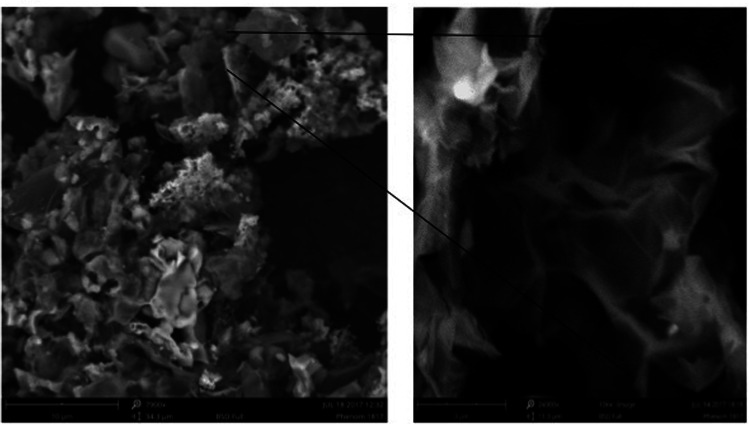
Scanning electron microscopy images of GO (graphene oxide) and GO-NH_2_-(PP-PPO) (amine-functionalized graphene oxide partially purified polyphenol oxidase enzyme) complex.

Fig. 5a presents the scanning electron microscopy images of GO and GO-NH_2_-PPO. The SEM image of GO indicates sheet-like morphology. The sheet-like morphology of GO indicates the chemical exfoliation of graphite, and the addition of oxygen functional groups in between the layers and the flower-like mass may represent the immobilized enzyme.

Zeta potential indicates the stability of colloidal solutions.^46^ The magnitude of zeta potential designates the degree of electrostatic repulsion in similarly charged surfaces of a material dispersed in a solvent. GO-NH_2_ exhibited a zeta potential ∼9.25 times higher than that of GO, whereas GO-NH_2_-(PP-PPO) exhibited ∼5.45 times higher zeta potential than GO-NH_2_ (Table 1). The results indicated that GO-NH_2_ had greater stability of colloidal dispersion than GO, and when immobilized with PP-PPO, the stability increased further. The high colloidal stability of PP-PPO-immobilized GO-NH_2_ is consistent with degradation as the experiments are mostly carried out in liquid media.

**Table 1:** Zeta potential analysis of GO, GO-NH_2_ and GO-NH_2_-(PP-PPO) complex

Sample	Zeta potential (mV)	Zeta deviation (mV)	Conductivity (mS cm^−1^)
GO	−0.595	183	0.0479
GO-NH_2_	−5.50	15.2	0.174
GO-NH_2_-(PP-PPO)	−20	8.51	0.0394

**Fig. 6:**
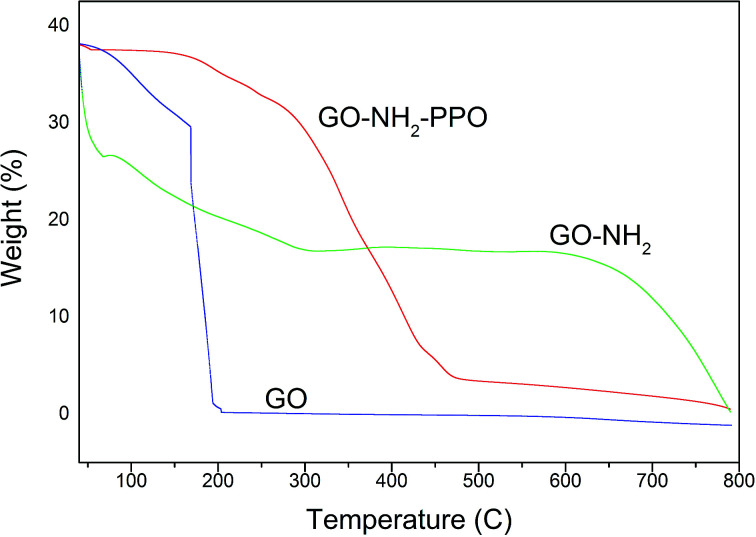
Thermogravimetric analysis (TGA) of GO (graphene oxide), GO-NH_2_ (amine-functionalized graphene oxide), and GO-NH_2_-(PP-PPO) (amine-functionalized graphene oxide complex).

Fig. 6 presents the thermogravimetric analysis (TGA) of the samples GO and GO-NH_2_. GO was thermally unstable and upon heating, weight loss was observed. The initial weight loss of 22% at 169 °C was ascribed to the removal of trapped water molecules and epoxy oxygen functional groups. Major weight loss of 74% observed at 200 °C may be due to the removal of labile oxygen functional groups by pyrolysis, yielding CO, CO_2_, and steam.^47,48^ Huge weight loss due to the oxygen atoms indicates high degree of oxidation during chemical exfoliation of graphite. Functionalization of GO by amine groups enhances the thermal stability of GO. Below 100 °C, GO-NH_2_ shows nearly zero weight loss.^49^ Weight loss of 87.5% occurs gradually from 100 to 475 °C. This observation also indicates that oxygen functional groups have been replaced by amine functional groups during Bucherer's reaction. A weight loss of 23% observed below 100 °C in PP-PPO-immobilized GO-NH_2_ might be due to the moisture content of the enzyme. The TGA curve of GO-NH_2_-(PP-PPO) shows 50% weight loss within 400 °C, which might be due to the loss of functionalized amine groups and organic residues of the enzyme.

At 750 °C, 67% weight loss was observed. The weight loss in GO-NH_2_-(PP-PPO) was lower than those of GO and GO-NH_2_, where 99.1% and 93.1% weight losses occurred at 750 °C. The possible reason might be the presence of copper in catalytic sites in PPO, which is not lost on heating to 750 °C and adds to the weight of the pan in TGA.

### Phenol degradation experiments

The activity of PPO in GO-NH_2_-(PP-PPO) complex was evaluated and compared with that of the free enzyme to determine the loading capacity of PP-PPO on to GO-NH_2_. The loading efficiency of PP-PPO on to GO-NH_2_ was calculated to be 81%. The experiments with GO and GO-NH_2_ on phenol removal demonstrated no changes in phenol concentration and hence were not included in the experimental studies. Furthermore, the initial trial experiments using GO-NH_2_-(PP-PPO) complex with 500 ppm phenol displayed complete removal within 3 h, whereas for the same concentration of free enzyme, phenol could not be removed completely even after 24 h incubation. This result has been ascribed to the inhibition of free enzyme activity due to the intermediates released during degradation. Zhang *et al.*^43^ reported higher degradation efficiency for immobilized enzymes compared to that of free enzymes and stated that immobilized enzymes are more suitable for degradation experiments. The possible reason might be the resistance of the immobilized enzyme towards higher concentration of a pollutant compared to that of free enzymes. The immobilization matrix GO selectively improves the catalytic activity of some enzymes and does not affect the activity of others.^50^

**Fig. 7:**
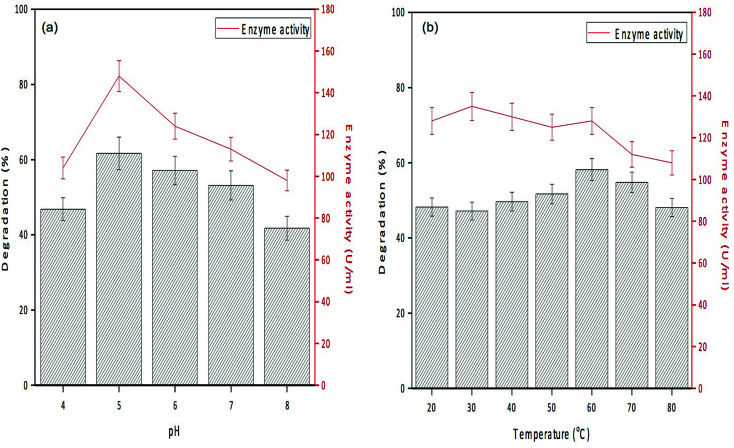
Degradation efficiency and enzyme activity profile of GO-NH_2_-(PP-PPO) (amine-functionalized graphene oxide complex) at (a) different pHs and (b) different temperatures.

Fig. 7a depicts the enzyme activity profile of the immobilized enzyme studied at different pH conditions, and it revealed that the maximum activity (148 U mL^−1^) was observed at pH 5.0. With reference to temperature, the immobilized enzyme displayed maximum activity at 30 °C (Fig. 7b). Similar to these observations, phenol degradation efficiency of the complex at varied pH conditions showed 46–61% degradation at pH of 4.0–5.0 and at the temperature of 30 °C. With respect to stability of the enzyme, the immobilized enzyme was stable at wide pH (4.0–7.0) and temperature (30–45 °C) conditions, whereas PPO isolated from litchi pericarp showed inactivation at higher pH and temperature.^51^

**Fig. 8:**
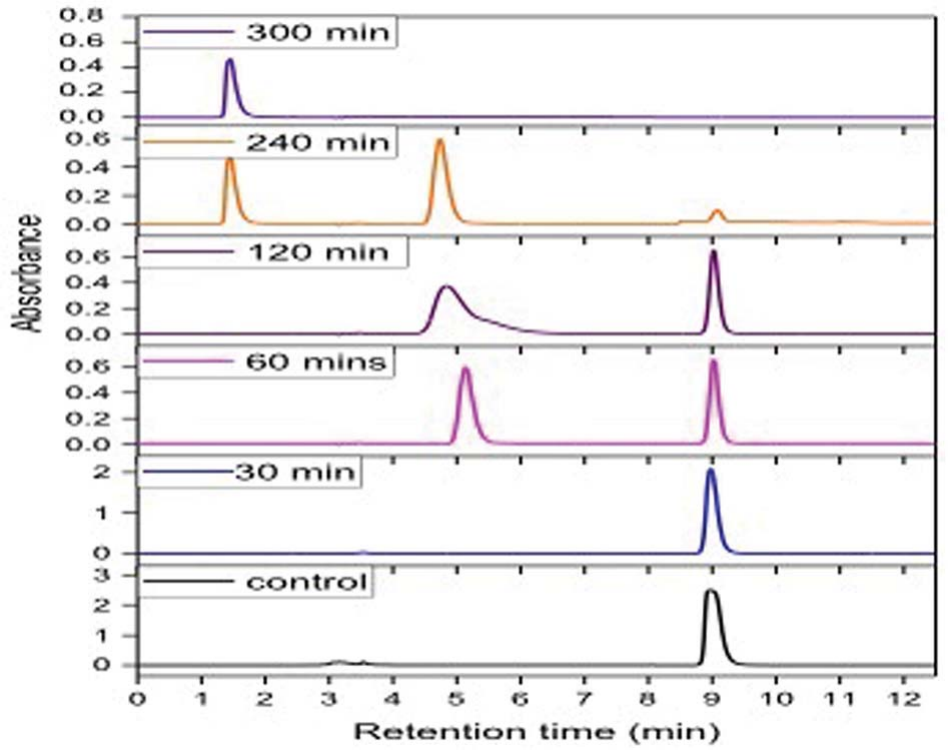
HPLC chromatogram of phenol at 1000 ppm concentration at different time intervals of incubation with GO-NH_2_-(PP-PPO) (amine-functionalized graphene oxide complex).

Degradation experiments were carried out for 1000 ppm phenol at the optimum conditions of pH (5.0) and temperature (30 °C), and samples were withdrawn every 15 min up to 1 h and at every hour thereafter. Fig. 8 presents the HPLC graph of degradation of phenol using GO-NH_2_-(PP-PPO) along with the standard at 1000 ppm concentration. It has been observed that over time, the intensity of the peak observed at RT 9.0 corresponding to phenol decreases, which indicates the degradation of phenol. The degradation efficiency increased with time from 40% in 15 min to 99.5% in 300 min. After 5 h of incubation with GO-NH_2_-(PP-PPO) complex, complete transformation was observed. Furthermore, HPLC results showed additional peaks at RT of 5.1 and RT of 1.4, corresponding to the intermediate products formed during the degradation process (Fig. 8). On comparing the intermediate peaks, RT of 5.1 corresponds to catechol and RT of 1.4 corresponds to hydroquinone.

### Reusability and storage stability

**Fig. 9:**
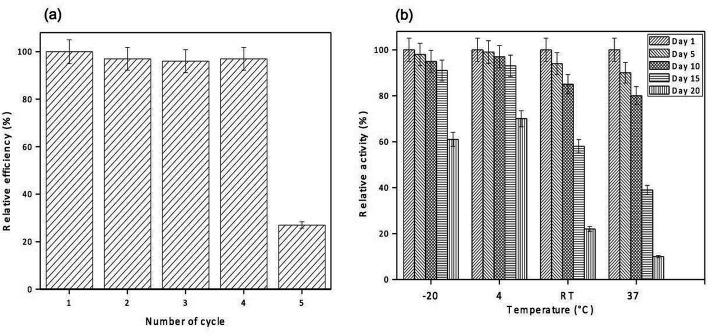
(a) Reusability and degradation efficiency of GO-NH_2_-(PP-PPO) (amine-functionalized graphene oxide partially purified polyphenol oxidase enzyme) complex with reference to phenol degradation. (b) Storage stability of immobilized enzyme (GO-NH_2_-(PP-PPO)) (amine-functionalized graphene oxide partially purified polyphenol oxidase enzyme) complex with reference to four different temperatures (−20, 4, room temperature (RT) and 37 °C) and the relative activity.

The reusability of GO-NH_2_-(PP-PPO) complex for phenol degradation was analyzed by measuring the degradation efficiency of the complex after each cycle. After each experimental cycle, the samples were analyzed using HPLC to assess degradation. The reusability studies with the complex revealed that the complex retained >98% of its activity up to 5 cycles, <85% after 7 cycles and <50% after 9 cycles of reuse (Fig. 9a). The results indicated that the complex was reusable before losing its degradation efficiency and activity. The loss of activity might be due to repeated washing.^29^

With respect to storage stability, the activity of free enzyme was completely lost within 3 days at all the temperatures studied. However, immobilized enzyme retained more than 90% of its activity until 15 days of incubation at temperatures of −20 °C and 4 °C. At room temperature and 37 °C, only 85% and 80% of activities were retained up to 10 days, respectively, and loss in the activity was observed after 10 days (Fig. 9b).

Table 2 depicts the comparative efficacy of phenol degradation studies using immobilized enzymes in different matrices. Among all the matrices, amine-functionalized graphene displayed appreciable degradation efficiency with increase in reusability cycle as well as stability of the immobilized enzymes. The complex prepared in the present study removes phenol at a much faster rate and can be effectively used for transformation of phenolic compounds at industrial scale.

**Table 2:** Comparison of degradation efficiency and reusability of immobilized enzymes for phenolic compounds

Compound	Enzyme	Matrix	No. of cycles	Time taken (h)	Concentration (ppm)
Phenol^21^	Laccase	Silica nanoparticles	10	3	50–100
Phenol^52^	Tyrosinase	Sodium alumino-silicate	4	0.33	100
Phenol^53^	HRP	GO	4	3	90
Chlorophenol^54^	PPO	Fe_3_O_4_/GO	4	3.5	4.7
Phenol^55^	HRP	Carbon nanospheres	5	1.25	161.68
Phenol^15^	HRP	Magnetic beads	—	0.25	188
Present study	PPO	GO-NH_2_	5	5	1000

## Conclusion

The present study explores whether amine-functionalized graphene oxide (GO) and phenoloxidase complex can perform the removal of phenol from the aqueous phase. Also, the reusability of the complex for the maximum number of cycles was tested since it can appreciably reduce the cost involved in the direct use of enzymes for environmental cleanup. The first step involves amine functionalization of GO, followed by preparation of the complex using polyphenol oxidase extracted from *Solanum tuberosum*. The enzyme was partially purified before forming a complex with amine-functionalized GO. Various analytical techniques such as UV, XRD, FTIR, Raman, zeta potential and TGA were performed to authenticate the complex formation. The surface morphology of GO analyzed using SEM revealed sheet-like morphology before complexation with PPO, whereas the complex showed aggregates, which could be due to the presence of enzyme. The results on optimization studies revealed that pH of 5.0 and temperature of 30 °C are the optimum conditions for degradation of phenol, where the enzyme exhibits higher activity compared to enzymes studied in other conditions. Under optimum conditions, the GO-NH_2_-(PP-PPO) complex could completely degrade 1000 ppm of phenol, as inferred from HPLC analysis with catechol and hydroquinone as intermediates. The GO-NH_2_-(PP-PPO) complex retained its >98% activity up to 5 cycles, <80% after 7 cycles and <50% after 9 cycles of reuse. With respect to storage stability, >90% activity was retained after 15 days of storage at −20 °C and 4 °C, and >90% activity was retained only up to 10 days when stored at room temperature and 37 °C.

## Funding source

No external funds were used for the study.

## Conflicts of interest

There are no conflicts to declare.

## Supplementary Material
